# Forensic features and phylogenetic structure survey of four populations from southwest China *via* the autosomal insertion/deletion markers

**DOI:** 10.1093/fsr/owad052

**Published:** 2024-01-16

**Authors:** Han Zhang, Meiqing Yang, Hongling Zhang, Zheng Ren, Qiyan Wang, Yubo Liu, Xiaoye Jin, Jingyan Ji, Yuhang Feng, Changsheng Cai, Qianchong Ran, Chengtao Li, Jiang Huang

**Affiliations:** Department of Forensic Medicine, Guizhou Medical University, Guiyang, Guizhou, China; Institute of Forensic Science, Fudan University, Shanghai, China; Department of Forensic Medicine, Guizhou Medical University, Guiyang, Guizhou, China; Department of Forensic Medicine, Guizhou Medical University, Guiyang, Guizhou, China; Department of Forensic Medicine, Guizhou Medical University, Guiyang, Guizhou, China; Department of Forensic Medicine, Guizhou Medical University, Guiyang, Guizhou, China; Department of Forensic Medicine, Guizhou Medical University, Guiyang, Guizhou, China; Department of Forensic Medicine, Guizhou Medical University, Guiyang, Guizhou, China; Department of Forensic Medicine, Guizhou Medical University, Guiyang, Guizhou, China; Department of Forensic Medicine, Guizhou Medical University, Guiyang, Guizhou, China; Department of Forensic Medicine, Guizhou Medical University, Guiyang, Guizhou, China; Department of Forensic Medicine, Guizhou Medical University, Guiyang, Guizhou, China; Institute of Forensic Science, Fudan University, Shanghai, China; The Key Laboratory of Environmental Pollution Monitoring and Disease Control, Ministry of Education, Guizhou Medical University, Guiyang, China

**Keywords:** InDels, Guizhou people, phylogenetic structure, AGCU InDel 50 kit, forensic genetics

## Abstract

Insertion/Deletion (InDel) polymorphisms, characterized by their smaller amplicons, reduced mutation rates, and compatibility with the prevalent capillary electrophoresis (CE) platforms in forensic laboratories, significantly contribute to the advancement and application of genetic analysis. Guizhou province in China serves as an important region for investigating the genetic structure, ethnic group origins, and human evolution. However, DNA data and the sampling of present-day populations are lacking, especially about the InDel markers. Here, we reported data on 47 autosomal InDels from 592 individuals from four populations in Guizhou (Han, Dong, Yi, and Chuanqing). Genotyping was performed with the AGCU InDel 50 kit to evaluate their utility for forensic purposes and to explore the population genetic structure. Our findings showed no significant deviations from Hardy-Weinberg and linkage equilibriums. The combined power of discrimination (CPD) and the combined power of exclusion (CPE) for each population demonstrated that the kit could be applied to forensic individual identification and was an effective supplement for parentage testing. Genetic structure analyses, including principal component analysis, multidimensional scaling, genetic distance calculation, STRUCTURE, and phylogenetic analysis, highlighted that the genetic proximity of the studied populations correlates with linguistic, geographical, and cultural factors. The observed genetic variances within four research populations were less pronounced than those discerned between populations across different regions. Notably, the Guizhou Han, Dong, and Chuanqing populations showed closer genetic affiliations with linguistically similar groups than the Guizhou Yi. These results underscore the potential of InDel markers in forensic science and provide insights into the genetic landscape and human evolution in multi-ethnic regions like Guizhou.

**Key points:**

- InDel markers show promise for forensic individual identification and parentage testing via the AGCU InDel 50 kit.
- Genetic analysis of Guizhou populations reveals correlations with linguistic, geographical, and cultural factors.
- Guizhou Han, Dong, and Chuanqing populations showed closer genetic affiliations with linguistically similar groups than the Guizhou Yi.

## Introduction

Guizhou, in Southwest China, is a landlocked province located on the western edge of Yungui Plateau; it is a source of rich human genetic, cultural, ethnolinguistic, and unique geographical resources. Demographically, it is one of China’s most diverse provinces. Minority groups account for more than 36% (Seventh National Census of China) of the population, with 18 native minorities (these include Miao, Dong, Yi, Bouyei, and Sui). Since ancient times, Guizhou has been an essential transportation hub on the Silk Road; it is also a conduit and corridor for cultural exchange and integration of various nationalities in southwest China [1]. Ancestors of all nationalities from all over China congregate there. As the only province in China with no plain support and dominated by mountains and hills, complex topography affects the communication between different populations. This can have severe genetic consequences. It is also reflected in the linguistic diversity of Guizhou populations, research indicates a broad association between genetic similarity and linguistic affiliation [2]. Previous studies have demonstrated that unique genetic ancestral components are present in modern populations under isolated and remote geographical conditions, such as the Kalash [3], Guangxi Miao [4], Hainan Li ethnic groups [5], and the Andamanese [6]. Our team’s previous studies also found evidence of the unique genetic ancestry admixture and complex population genetic history in Guizhou populations, as well as long-term genetic stability in the Yungui Plateau [7–11]. Due to the potential genetic substructure, allele frequency data are necessary to correctly calculate kinship and the forensic strength of evidence for the investigated population groups. Therefore, it is important to collect and analyze relevant data on the Guizhou populations before the development and application of new forensic identification systems.

An insertion/deletion (InDel) is a genetic marker with great application potential and several advantages: it is a length-based polymorphism that can have compatibility with current capillary electrophoresis platform, with a lower mutation rate, the absence of a stutter peak, widespread distribution throughout the genome, and smaller amplicon sizes. InDels combine the common desirable features of both short tandem repeats (STRs) and single nucleotide polymorphisms (SNPs) [12, 13]. Especially, InDel avoids the stutter bands that complicate STR profile interpretation of mixtures. In addition, because of the small amplicon size, it is an excellent candidate for examining corrupted and degraded material in the event of STR typing failure [14, 15]. STR polymorphisms are recognized as the gold standard in forensic individual identification and parentage testing. Therefore, laboratories around the world have established platforms for STR analysis [16]. InDels and STRs are both length polymorphism genetic markers that can share an analysis platform of polymerase chain reaction (PCR) and capillary electrophoresis. Thus, InDels have the advantage that the globally established hardware can be used for their analysis.

In 2002, Weber et al. [17] first reported 2000 InDel loci at the genome-wide level. The 1000 Genomes Project increased the number of loci to 3.6 million [18]. In 2020, Bergström et al. [19] considerably extended the number of InDels to 8.8 million. In the latest report from Cell 2022, Byrska-Bishop et al. [20] presented results from high-coverage whole-genome sequencing (WGS) of the expanded 1 kGP cohort comprising 14 435 076 InDels, which is freely available to the research community. This gives us sufficient candidate loci to choose from to construct a consistent amplification system for different forensic purposes. For example, previous studies have confirmed that we can use the frequency divergence of InDels allele to distinguish between geographically or linguistically different populations, and InDels can be used as an ancestry-informative marker (AIM) [21–23].

The first commercial InDel kit, the Investigator DIPplex kit, was evaluated in 2011 [24]. It has been studied in more than 100 populations around the world for more than a decade [25–27]. Our laboratory has also completed data collection and analysis of the 30-InDel system in seven populations in Guizhou province. Confirming that the Investigator DIPplex kit can be used for forensic investigations in the Guizhou populations, the studied Guizhou groups retain close genetic affinity with geographically and linguistically close populations [11, 28–30]. In 2019, Chinese forensic scholars Chen et al. [15] developed the AGCU InDel 50 kit comprising 47 autosomal InDel loci (completely different from Investigator DIPplex kit), 2 Y chromosome InDel loci, and amelogenin, with a higher discriminatory power and more evenly distributed allele frequencies in the Chinese population than those based on 30 InDels contained in Investigator DIPplex kit. However, to date, only one piece of population data (Zunyi Gelao, AGCU InDel 50 kit) has been released for the Guizhou region [31]. This is extremely unhelpful for the construction of the Guizhou population database and the promotion and application of InDel genetic markers in Guizhou. Thus, we present comprehensive analyses, based on 47 A-InDels included in the AGCU InDel 50 kit, of 591 samples from four Guizhou populations (Han, Dong, Yi, and Chuanqing) belonging to the Sinitic, Tai-Kadai, and Tibeto-Burman language groups.

Dong and Yi are native to Guizhou, and the Dong population is larger in Guizhou than in any other province. In particular, Guizhou is the location of the largest Dong minority village in China, Zhaoxing, which is also called “No. 1 Dong Village”. Arguably, Guizhou is one of the best places to investigate the genetic resources of the Dong ethnic group. Research has shown that Yi people from different regions have significant genetic differences [32]; we may also find clues in their rich and varied Yi dialects, such as Nuosu, Lalo, Lolopo, Nisu, Sani, and Nasu [33]. As much data as possible should be collected from Yi people all over the country. Chuanqing is the largest unrecognized ethnic group in Guizhou. It comprises ~700 000 people according to the data of the Seventh National Census of China (2021), who speak a Sinitic language. The origin of the Chuanqing people has always been disputed as to whether they were Han Chinese or indigenous people of southwest China. Although research has shown that Chuanqing samples are genetically similar to the southern Han Chinese [34], we still believe that sufficient data on Chuanqing should be collected to complement the genetic material of the Guizhou Han population. The Han Chinese comprises over 90% of China’s population. Chen et al. [35] proved the existence of genetic substructure in Han Chinese populations with the main pattern a “north–south” cline. Guizhou’s mountainous terrain and inter-cultural restrictions may lead to more complex genetic consequences. Our study aimed to provide further population reference data from different geographic and linguistic populations, calculate forensic parameters, and explore the phylogenetic relationships and population structure. Consequently, our study enriches the population database of InDels in southwest China.

## Materials and methods

### Sample preparation, DNA extraction, and ethics statement

Our study’s purpose and sample collection were approved by the Ethics Committee of Guizhou Medical University (approval number: XDYX2019009) and conducted under the standards of the revised Helsinki Declaration of 2013 [36]. After receiving written informed consent, blood samples were collected from 592 unrelated healthy individuals born in Guizhou province, southwestern China, comprising 150 Dong, 153 Yi, 198 Han, and 91 Chuanqing individuals. Strict screening criteria were applied for all of these participants: (i) self-reported healthy condition; (ii) no biological kinship related to anteriorly recruited participants within at least three generations. Our sample was sifted through thousands of paternity tests over the years. We checked the corresponding family records to ensure that the parents were in non-consanguineous marriages of the same ethnic group. Finally, we asked if there were any special circumstances that are not reflected in the certificate, thus ensuring the best possible reliability of the sample. Human genomic DNA was extracted using Chelex-100 (Bio-Rad, Hercules, CA, USA), quantified, and adjusted to 1.0–2.0 ng/mL for amplification.

### Reference dataset

We obtained the reference dataset in two ways downloaded from 1000 Genomes Phase III release [18] and collected from previously published articles [15, 31, 37–43]. Finally, we successfully constructed two datasets of allele frequency and raw genotype data, namely Dataset I and Dataset II. All the populations in Dataset II are included in Dataset I. Dataset I includes 7 541 individuals from 58 worldwide human populations. Dataset II is composed of 6 035 individuals from 49 worldwide populations. The detailed information for the reference populations is presented in Supplementary Table S1. It must be noted that Dataset II lacks genotypes for rs67939200. Thus, only 46 A-InDels were involved in the subsequent analysis relating to the genotype data.

### Amplification and genotyping

Multiplex PCR amplification was performed in a 12.5-mL reaction volume on a single PCR multiplex as recommended by the manufacturer for the AGCU InDel 50 kit (AGCU, Scien Tech Inc. Wuxi, China) [15] and conducted on a ProFlex 96-Well PCR System (Thermo Fisher Scientific, Lenexa, KS, USA). Amplification products were subsequently separated using capillary electrophoresis (36-cm capillary arrays) on a 3500XL Genetic Analyzer (Thermo Fisher Scientific) with the POP-4 polymer. Moreover, the genotyping of each InDel was performed by GeneMapper ID-X version 1.5 Software (Thermo Fisher Scientific). DNA 9948 and ddH_2_O were utilized as the positive control and negative control, respectively.

### Statistical analysis

We used Arlequin software v3.5 [18, 44] to calculate the Hardy–Weinberg equilibrium (HWE) as well as *P* values of linkage disequilibrium (LD) (number of permutations was 1 000) and calculated the observed heterozygosity (Ho) and expected heterozygosity (He). The visualization of LD based on *r*^2^ values between 47 InDels was performed using the SNPAnalyzer software v2.0 [45]. Subsequently, forensic statistics-related parameters, including allele frequency, match probability, probability of exclusion (PE), discrimination power (PD), typical paternity index (TPI), and polymorphism information content (PIC), were calculated using STR Analysis for Forensics (STRAF) online software [46]. We computed two genetic distances, *D*_A_ based on allele frequencies and *F*_st_ based on the genotype data, using PHYLIP v3.52 [47] and Genepop v4.0 [48], respectively. We then performed frequency-based and genotype-based principal component analysis (PCA) using Multivariate Statistical Package (MVSP) Software v3.22 [49] and STRAF [46]; we set the tolerance of eigenanalysis to 1E-007 when running the PCA. Furthermore, we performed multidimensional scaling (MDS) using IBM SPSS version 21.0 based on the *D*_A_ genetic distance matrix. Visualization was performed using R version 4.2.2 (https://www.r-project.org) to plot scatter plots and heatmaps. Neighbour-joining (N-J) phylogenetic tree reconstruction was performed using Molecular Evolutionary Genetics Analysis (MEGA) software v7.0 [50] using Nei’s and *D*_A_ genetic distance matrices based on two different population datasets composed of 58 groups and 49 groups. Finally, for the ancestry component composition, we used the raw genotype dataset to run STRUCTURE analysis in STRUCTURE version 2.3.4.21 [51] using the parameters of 10 000 burn-ins and 10 000 MCMC under the “LOCPRIOR” model, running 15 replicates from *K* = 2 to *K* = 8. Finally, we implemented the graphic programme of AncestryPainter [52] to illustrate the ancestry component compositions by running a Perl programme and an R script.

## Results

### Linkage disequilibrium and Hardy–Weinberg equilibrium testing of 47 A-InDels

Our study provided the newly obtained 591 individuals’ genotype data of 47 A-InDels in four Guizhou populations which is presented in Supplementary Table S2. We first performed HWE testing and LD analysis for the above data. No significant departures regarding the *P* values from HWE and LD were observed for the four studied populations after Bonferroni correction (Supplementary Tables S3 and S4). Furthermore, we calculated *r*^2^ values to measure the degree of linkage between pairwise InDel loci. The *r*^2^ values of the LD for the 47 InDel loci were illustrated in the form of heatmap, as shown in Supplementary Figure S1. The various degrees of red in the small square areas indicate the levels of linkage between loci. Based on the criterion of *r*^2^ <0.8, we did not find any linkage phenomenon between pairwise InDel loci for studied groups. The abovementioned results indicate that we can ensure the reliability of our study sample for evaluating larger groups, and that we can treat the 47 A-InDels as independent loci in the subsequent analysis.

### Forensic parameters and allele frequency distributions in 54 populations

**Figure 1:**
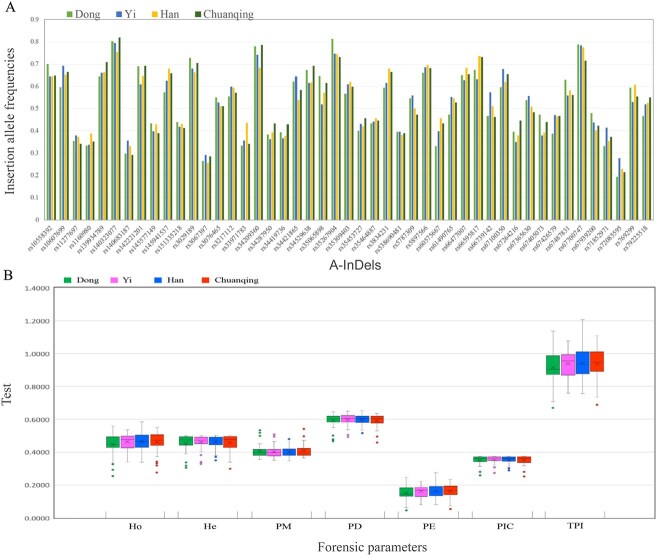
Combination diagram of insertion allele frequencies and forensic parameters of the 47 A-InDels. (A) Allele frequencies are shown by histogram; (B) Forensic parameters are displayed in the form of a boxplot. Ho: heterozygosity; He: heterozygosity; PM: matching probability; PD: discrimination power; PE: probability of exclusion; PIC: polymorphism information content; TPI: typical paternity index.

We are the first to calculate the forensic parameters and allele frequencies of 47 A-InDels in four Guizhou populations (Supplementary Table S3). Moreover, the insertion frequencies and forensic parameters are displayed in a combination of boxplot and histogram (Figure 1). Finally, the statistical analysis results show that the combined powers of discrimination (CPD) for the Han, Dong, Yi, and Chuanqing groups were 0.999 999 999 999 999 999 757 9, 0.999 999 999 999 999 999 644 9 and 0.999 999 999 999 999 999 778 7, 0.999 999 999 999 999 999 582 3, respectively, and the combined powers of exclusion (CPE) were 0.999 759 9, 0.999 562 5, 0.999 746 3, and 0.999 745 9, respectively.

**Figure 2:**
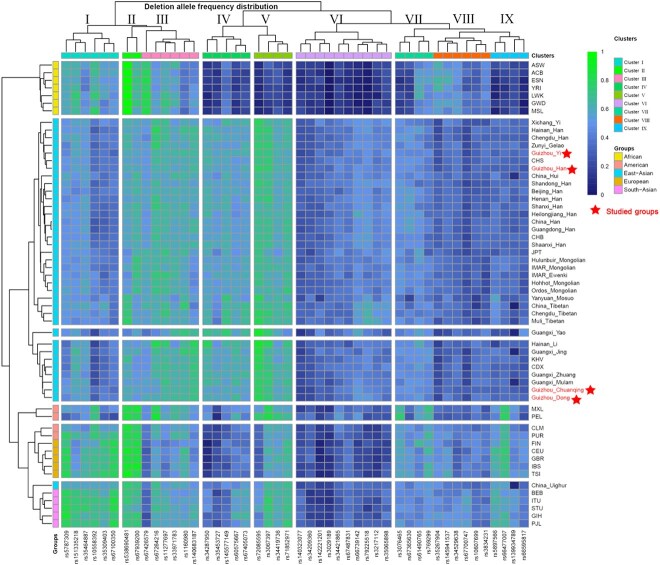
Heatmap of the 47 A-InDels deletion allele frequencies in the four studied populations and other 54 worldwide reference populations. All populations are divided into five groups at the continent level, and all loci are divided into nine clusters based on frequency distribution features.

To explore the discriminatory power of 47 A-InDels from the AGCU InDel 50 kit for different continental and linguistic populations, we constructed heatmaps based on deletion allele frequencies, as shown in Figure 2, including 26 populations from 1000 Genomes Phase III, and 28 groups from previously published articles; the details and references are given in Supplementary Table S3. Generally, the minor allele frequency (MAF) is ~0.3–0.5 in East Asian groups. The result is within our expectations because this is one of the screening criteria for loci used for forensic individual identification and parentage testing. However, the remarkable thing about the East Asian groups is that InDels included in Clusters II–V showed relatively higher deletion allele frequencies. In Clusters I and VI–IX, the frequency was lower. The frequency of 47 A-InDels showed significant divergences in African populations, followed by American and European groups. InDels located in Cluster III and Clusters VII–IX showed that the MAF was ~0.4–0.5 in American, European, and South Asian populations, indicating that these InDels can be chosen as candidate markers used to design forensic kits for individual identification in the corresponding populations. The InDel loci contained in Clusters IV–VI and IX exhibited low heterozygosity in American populations. InDel loci included in Clusters II and IV–V showed significant frequency diversity among populations from different continents, even for geographically and linguistically different populations from East Asia. This indicated that these loci have great potential to serve as useful AIMs for biogeographical ancestry inference. Moreover, we were surprised to find that rs139934789 in Cluster IX exhibited exceptionally low heterozygosity in China_Hui, China_Tibetan, Guangxi_Yao, Guangxi_Jing, Guangxi_Mulam, and China_Uighur from East Asia. Continental populations have clear clusters based on frequency difference, indicating that the combination of 47 A-InDel locus can be utilized as a complementary tool for population phylogenetic structure and biogeographical ancestry inference. Studied Guizhou populations are scattered in the East Asian cluster.

### Genetic structure and genetic affinity explorations

#### PCA

**Figure 3:**
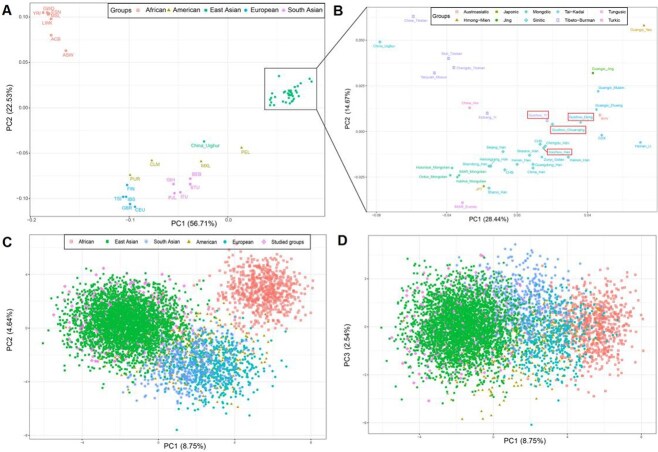
Principal component analysis (PCA) between the studied group and worldwide reference populations. (A) PCA plot of 58 populations from five continents constructed on the basis of 47 A-InDels allele frequencies. (B) PCA plot of 37 populations on the scale of East Asia constructed on the basis of allele frequencies. (C, D) PCA based on the genotype data of 46 InDel loci from 49 populations at the individual level.

To further exhibit the genetic background and relative relation between studied Guizhou groups and worldwide reference populations, we conducted frequency-based (Figure 3A and B) and genotype-based (Figure 3C and D) PCA at the group and individual levels. The first two PCs accounted for 79.24% (PC1 56.71%, PC2 22.53%) of the variances at the population level, revealing clear genetic clustering among the analyzed populations (Figure 3A). Among 47 A-InDels, rs10558392 (0.226) and rs538690481 (0.214) had the largest value of PCA variable loadings in PC1, whereas rs66477007 (0.381) and rs5897566 (0.347) had the largest value of PCA variable loadings in PC2. PCA variable loading describes the contribution of a component into a variable; if it is high (close to 1), the variable is well defined by that component alone. The maximum value of rs10558392 in PC1 shows that it has a high distinction between East Asian populations and the rest of the world. Details of the PCA variable loadings for 47 A-InDels are provided in Supplementary Table S7.

We could observe clear population clusters of African, European, South Asian, and East Asian populations; four groups from America (PUR, CLM, MXL, PEL) were poorly clustered. The four studied Guizhou groups clustered with East Asian populations in the upper right quadrant. However, the East Asian populations in this picture were too tightly clustered to be discernible, so we performed a separate analysis of these populations contained in the black box and China_Uighur (Figure 3B); linguistically different populations were labeled with different shapes and colours. PC1 accounted for 28.44% of the total variation, PC2 accounted for 14.67% of the total variation, rs10558392 (0.385) has the largest value of PCA variable loadings in PC1, and rs139934789 (0.514) has the largest value of PCA variable loadings in PC2 (Supplementary Table S7).

Four main genetic subclusters were observed within the East Asian populations: Mongolic, Sinitic, Tai-Kadai and Tibeto-Burman speakers. However, Guizhou_Yi did not cluster with linguistically close populations; it is closer to Sinitic-speaking populations. Obviously, Guangxi_Yao, China Uighur, and IMAR_Ewenki were located away from most of the East Asian populations. The studied groups were located in the region where Sinitic populations and Tai-Kadai populations connect, especially falling together with CHS, Chengdu_Han, Guangxi_Zhuang, KHV, and CDX. No significant genetic differentiation was observed among the studied Han, Dong, Yi, and Chuanqing populations. Moreover, in the genotype-based PCA (Figure 3C and D), we could not distinguish the studied populations clearly, as they overlapped with East Asian populations. However, we observed four large genetic clusters: African, European, East Asian, and South Asian populations.

#### Calculation of genetic distance

**Figure 4:**
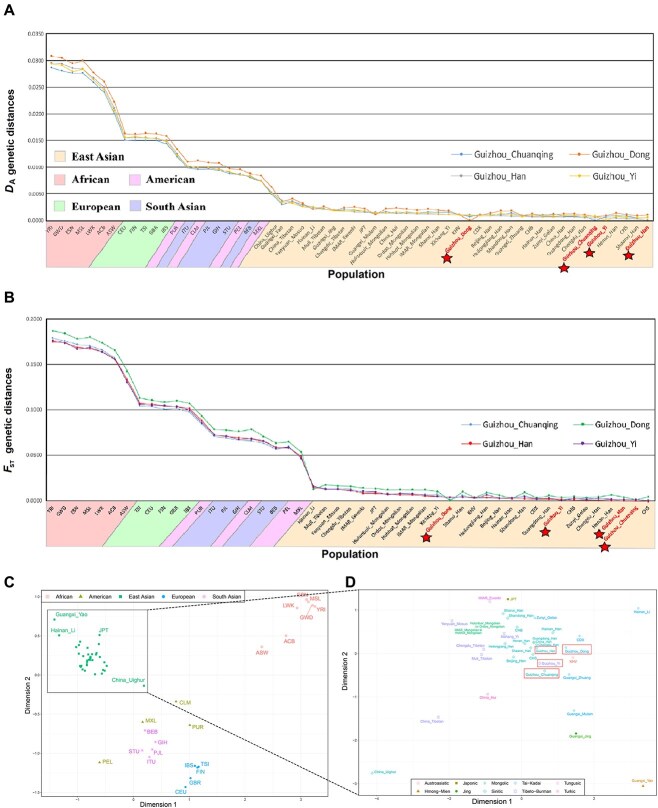
The genetic distance and multidimensional scaling between studied groups and reference populations on the continent scale. (A) Pairwise *D*_A_ genetic distances based on 47 A-InDels allele frequencies for Guizhou Han, Dong, Yi, and Chuanqing, and 54 global reference populations. (B) Pairwise *F*_st_ genetic distances based on 46 A-InDels genotype data for the studied groups and other 45 reference populations. (C) Multidimensional scalin (MDS) plots performed based on the *D*_A_ genetic distance, including 58 populations. (D) MDS plots performed on the scale of East Asia. MDS: multidimensional scaling.

In order to uncover the genetic similarity between the studied Guizhou groups and other reference populations more accurately, we calculated the frequency-based *D*_A_ genetic distance and genotype-based *F*_st_ genetic distance based on the data from Dataset I and Dataset II; these are presented in Figure 4A and B. The genetic distance matrix is shown in Supplementary Tables S5 and S6. The results were similar for *D*_A_ and *F*_st_; the genetic distance was greatest between the studied groups and the African population, followed by the European, the mixed cluster of American and South Asian populations, and the East Asian population. The difference in genetic distance values between the four studied groups and the reference East Asian populations was not significant, except for China_Uighur. We identified the three closest genetic relationship populations with each of our four studied groups based on *D*_A_ values: Guizhou_Han (CHS, 0.0003; Shaanxi_Han, 0.0003; Henan_Han, 0.0004); Guizhou_Dong (Guizhou_Chuanqing, 0.0006; Hainan_Han, 0.0007; Guangxi_Zhuang, 0.0008); Guizhou_Yi (CHS, 0.0005; Shaanxi_Han, 0.0005; Guizhou_Han, 0.0005); Guizhou_Chuanqing (Shaanxi_Han, 0.0005; Guizhou_Han, 0.0005; Guizhou_Dong, 0.0006).

Different types of genetic distances show slight differences in genetic relationship between the studied and other reference populations, especially among the East Asian population, perhaps because the genetic differences are too small among East Asian populations. The choice of algorithms and the types of data to be analyzed directly affects the measured genetic distance between studied populations and other reference populations. In addition, we conducted MDS based on *D*_A_ values to visualize and evaluate the results in more dimensions (Figure 4C and D). Four main clusters are clearly observed in the MDS scatterplot: the African, European, South Asian, and East Asian populations. All the studied Guizhou populations were close to each other and were consistent with geographical and linguistic classifications. Generally speaking, the results of *D*_A_, *F*_st_ genetic distances, and MDS are consistent with the patterns obtained in the previous frequency heatmap and PCA results.

#### Phylogenetic analysis

**Figure 5:**
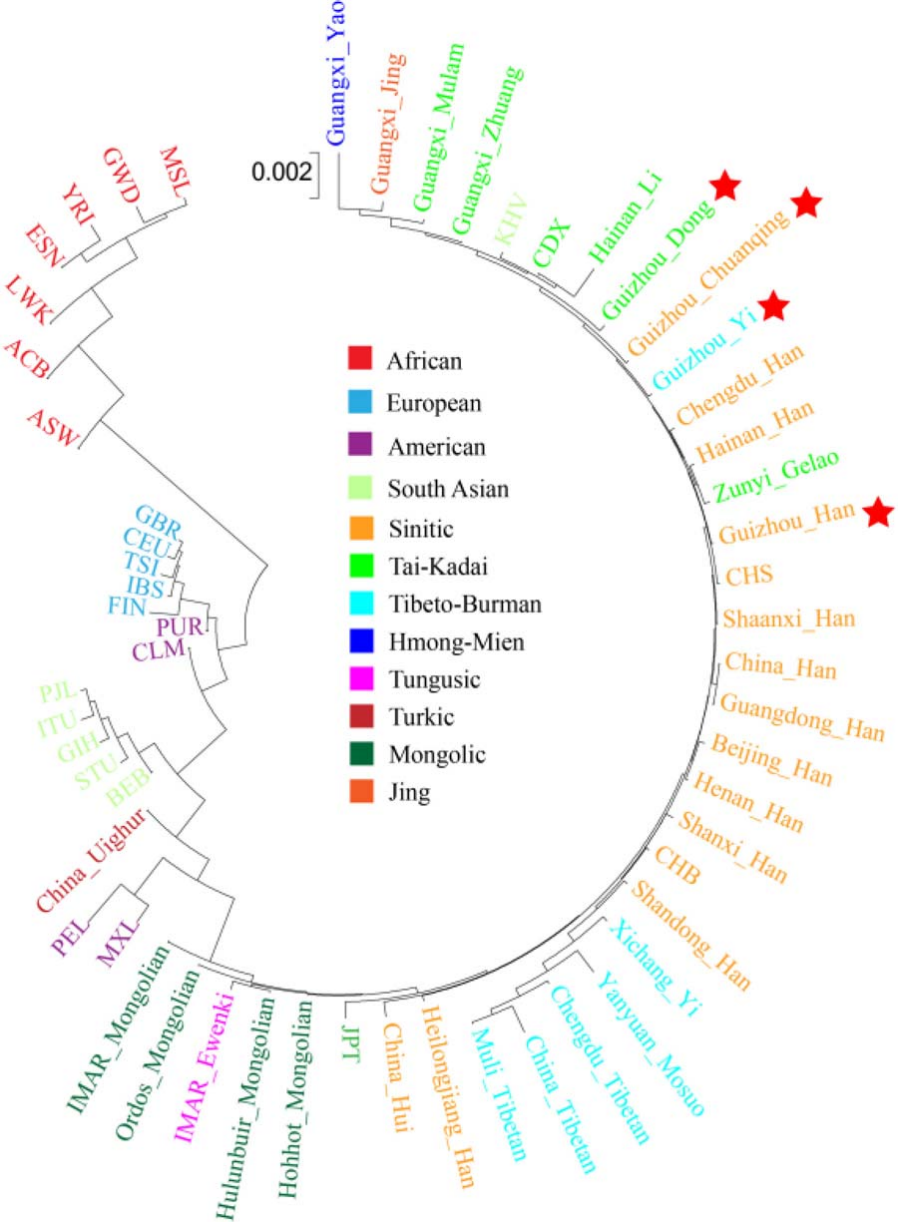
Phylogenetic relationships between our studied Guizhou populations and other 54 reference populations based on *D*_A_ genetic distances, construction of the phylogenetic tree by the neighbour-joining algorithm. The distance scale represents the number of differences between studied taxon and ancestor taxon.

To infer the phylogenetic relationships between the studied groups and reference populations, as shown in Figure 5, we constructed the N-J phylogenetic tree based on *D*_A_ genetic distances, including virtually the entire population data published so far on the AGCU InDel 50 kit. The results reveal two main clades: one clade contains only African populations, and the other includes all the European, American, South Asian, and East Asian populations. Subsequently, we continued to refine the subpopulations of East Asia based on linguistic and cultural differences. The Tai-Kadai-speaking, Sinitic-speaking, Tibeto-Burman-speaking, and Mongolian populations were clustered together. The four studied populations are scattered within the East Asian populations, and clustered tightly with geographically adjacent or linguistically related populations. Although it is noteworthy that China_Uighur, IMAR_Ewenki, and Guangxi_Yao have unique genetic variation compared with the other East Asian populations in our study, the genetic affinity between Guizhou_Yi and the language-related populations is weaker than that of other China’s ethnic minorities on the same macroscopic background. By macroscopic background, we mean the same reference populations, DNA data type, or analysis methods.

#### Population ancestry component analysis

To further model the ancestry composition and their corresponding admixture proportion of Han, Dong, Yi, and Chuanqing people residing in Guizhou province, we performed STRUCTURE analysis of genotypes in Dataset II, including 45 reference populations from five continents. We ran the hypothetical populations (*K*) from *K* = 2 to *K* = 8, as shown in Supplementary Figure S1. Simultaneously, we uploaded the results to the online website STRUCTURE HARVESTER (https://taylor0.biology.ucla.edu/structureHarvester) and obtained the optimum *K* value of *K* = 4. At *K* = 2, we found two main ancestral components, one from African, which is also present in large proportions in European, American, and South Asian populations; the other found mainly in East Asian populations. At *K* = 3, a new ancestral component has been identified, which belongs primarily to the European, American, and South Asian populations, although they remain essentially indistinguishable. At *K* = 4, the ancestral component, presented in purple, was found in East Asian people. No significant population substructure was observed within the East Asian population, including the four studied Guizhou groups. In addition, we implemented the programme of AncestryPainter, which uses a circular method to display the individuals’ ancestry (*K* = 4).

**Figure 6:**
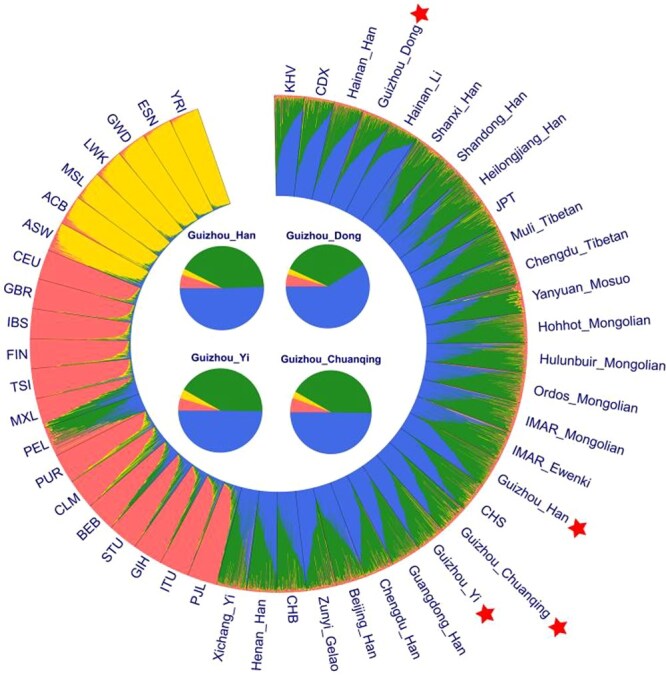
STRUCTURE analysis at individual level based on the Dataset II of four studied and 45 reference worldwide populations (the optimal *K* value was 4). Illustration of the ancestry component compositions by using the graphic programme of AncestryPainter. The pie charts were added in the center of the circular graph to highlight the target population.

The ancestry composition shown in Figure 6 was consistent with those described previously. The pie charts were added to the center of the circular graph to highlight the target population of Han, Dong, Yi, and Chuanqing. We found that the proportion of Han and Chuanqing ancestry was consistent; however, the ancestral component represented by blue was more abundant in Dong and other Tai-Kadai populations, and that represented by yellow (mainly from African populations) was more abundant in Yi compared with the other studied populations. As *K* increased (Supplementary Figure S1), no new genetic structural features of the population were found based on the proportion of ancestral components. However, it should be emphasized that the proportion of ancestral components in Hainan_Li was different from that of other East Asian populations; the ancestral components indicated by blue (Figure 6) and forest green (Supplementary Figure S1) were at a higher proportion in Hainan_Li. KHV, CDX, Hainan_Han, and the studied Guizhou_Dong are more similar in composition to Hainan_Li than in other ethnic groups with more southern East Asian ancestry. Hainan Island is the second-largest island administered by China. The Li people are suggested to be descendants of the earliest settlers of Hainan Island. Li people have lived a rather isolated lifestyle, with limited genetic admixture with surrounding populations [4, 53]. Therefore, Hainan Li has a close genetic affinity with ancestral Tai-Kadai-speaking populations.

## Discussion

There are various ethnic groups and lineages in southwest China. It is therefore a key area for the study of the origin, migration, and diversification of ethnic groups, as well as for the investigation of genetic resources. In particular, the study of the Guizhou population has attracted numerous scholars in terms of interest and importance [34, 54–57]. We therefore selected four populations from the Guizhou province with different characteristics in terms of the number, genetic origin, and cultural background to study, namely, Han, Dong, Yi, and Chuanqing. InDels are considered to be a molecular tool with great potential for forensic DNA profiling and practical applications after STR. We had previously completed genotyping and data analysis of seven populations in Guizhou based on the first commercial InDel kit, the Investigator DIPplex kit [11, 28–30]. In this study, we reported the latest 47 A-InDels data from 592 samples, obtained from the new commercial AGCU InDel 50 kit. The aim of our study was to provide Guizhou populations reference data for the application of InDel genetic markers, and to help illuminate the questions about the extent and structure of genetic variation in Guizhou populations.

First, no significant deviations were observed in the calculation of the HWE and LD for 47 A-InDels in the four investigated Guizhou groups, demonstrating that our sample and that data were representative; thus, the subsequent analysis can treat these loci as independent markers. The values of CPD were greater than 0.999 9 in all studied groups, confirming that the kit has sufficient forensic identification power. However, given that the values of CPE were relatively low (0.999 5–0.999 7), this kit might only serve as a complementary tool for paternity tests. Generally, the values of CPD, CPE, PIC, Ho, and He are higher than those from our previous studies obtained based on Investigator DIPplex kit. In particular, the lowest values of the forensic parameters showed a significant increase, but were still much lower than STR for the diallelic genetic markers [58, 59]. In addition, the MAF of the majority of the InDels in East Asian populations, including the four studied groups, is ~0.3–0.5, indicating that they are highly informative for use in forensic investigation.

We constructed heatmaps of deletion allele frequencies based on 47 A-InDels for the studied Guizhou groups and worldwide reference populations. We found significant frequency differences between populations on different continents. In particular, the InDels in Clusters II–V present more significant frequency differences among populations, suggesting that these loci with high population discrimination effectiveness can be adopted as AIMs. In addition, InDels in Cluster III and Clusters VII–IX displayed allele frequencies fluctuating at ~0.5 within East Asian, American, European, and South Asian populations. These could be selected as better candidates for individual identification on a global level. At the population level, we conducted genetic structure analysis through allele frequency (Dataset I) and genotype data (Dataset II). We found that the results of frequency-based PCA, *D*_A_ genetic distances, the MDS, and N-J phylogenetic tree performed based on *D*_A_ matrix were basically the same. We presented four obvious intercontinental clusters: African, European, East Asian, and South Asian clusters, but the American populations were poorly clustered. We also observed five blurry subclusters within the East Asian populations: Sinitic, Tai-Kadai, Tibeto-Burman, and Mongolic. Generally, this result is also consistent with previous studies by scholars that geographically, linguistically, and culturally similar populations have closer genetic affinities [7, 18, 60].

Our results for the four Guizhou groups conform to the genetic association described above. Chuanqing people clustered with Guizhou Dong and Yi, and had the closest *D*_A_ genetic distance to Guizhou Han and CHS, as well as the closest *F*_st_ genetic distance with Shaanxi Han and Guizhou Han. Considering all these results together, we are inclined to agree with the hypothesis that the Chuanqing people are Han Chinese who migrated to the Guizhou region through the form of military immigration [34]. The large genetic diversity between the Guizhou and Xichang Yi, as well as other Tibeto-Burman populations, should be noted. We may be able to explain this in terms of language, as the branches of Yi were multitudinous and collectively called Yi in the 1950s, with different lineages of Yi people speaking various Loloish languages, closely related to Burmese. Official authorities recognize as many as six Yi languages that are mutually unintelligible. Most of the Yi people in Guizhou speak the Nasu language, whereas most of the Yi people in Sichuan speak the Nuosu language. This could indicate different genetic origins for Yi people from different regions. We found that the Guizhou Han and Dong people were more closely related to people of the same language families and ethnicities. In addition, the results of genotype-based PCA, *F*_st_ genetic distances, and STRUCTURE analysis were consistent with the conclusions of the above analysis.

We were unable to find new population substructures in the studied population and other reference populations. The genetic ancestral components of the East Asian populations, including the investigated groups, are essentially the same. In summary, our study provides the latest InDel data for scientists and an extensive assessment of the application efficacy in Guizhou population. In terms of the resource itself, the limitation is its fragment length analysis and small amount of data; hence, more large-scale genome-wide sequencing projects should be performed in Guizhou.

## Conclusion

In this study, we present the first batch of genotype data and newest research results of 47 A-InDels based on the AGCU InDel 50 kit from 592 samples from Han, Dong, Yi, and Chuanqing people in Guizhou. We successfully constructed allele frequency Dataset I (58 populations and 7 541 samples) and genotype data Dataset II (49 populations and 5 742 samples). The forensic parameter statistics verified that the AGCU InDel 50 kit could be used for forensic individual identification, but only as a complementary tool for paternity tests. Our findings in the allele frequency distribution from worldwide populations indicated that 11 and 20 out of the 47 A-InDels could be selected as candidates for biogeographical ancestry inference at the continental level. The PCA, MDS, genetic distances, phylogenetic tree, and STRUCTURE analyses indicate that the genetic structure of the studied groups is consistent with the pattern of geographically, linguistically, and culturally close populations that are genetically close to each other. In conclusion, further genomic studies should be performed in Guizhou province in southwest China, such as whole-genome sequencing and ancient DNA study, to provide high-coverage human genome-wide data and explore the deep population history and genetic variation.

## Supplementary Material

Supplementary_owad052
