# Supplementary material for: Forensic features and phylogenetic structure survey of four populations from southwest China via the autosomal insertion/deletion markers
Source: Forensic Sci Res. 2024 Jan 16;9(2):owad052. doi: 10.1093/fsr/owad052 (PMC11102079; doi:10.1093/fsr/owad052)
Supplement: Supplementary_owad052 [file supplementary_owad052.docx]

**Supplementary Table legends**

**Table S1.** Detailed information on the populations involved in the present study.

**Table S2.** Genotype data of the AGCU InDel 50 kit in four studied Guizhou populations.

**Table S3.** Forensic parameters and *P* values of Hardy–Weinberg equilibrium in the studied populations.

**Table S4.** The p values of linkage disequilibrium in the studied populations.

**Table S5.** Pairwise DA genetic distances between the studied populations and 54 worldwide populations on the basis of the 47 InDels.

**Table S6.** Fst values between the studied people and other reference populations.

**Table S7.** PCA variable loadings of 47 InDels from the principal component analysis of 58 worldwide reference populations and 37 East Asia populations on the basis of allele frequency data.

**Supplementary Figure legends**

**
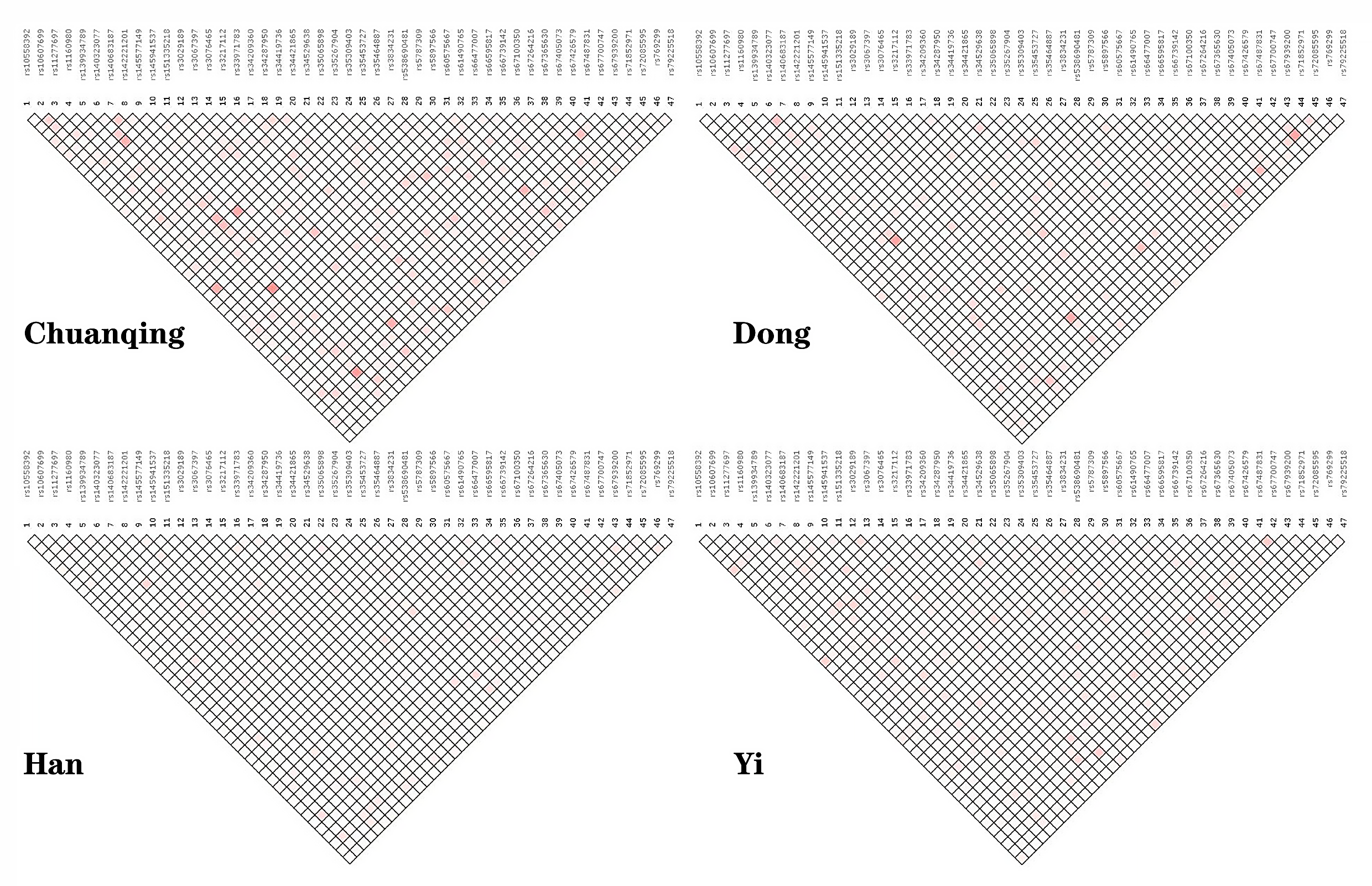
**

**Figure S1.** The linkage disequilibrium analysis schema among the 47 InDel loci of Han, Dong, Yi, and Chuanqing.


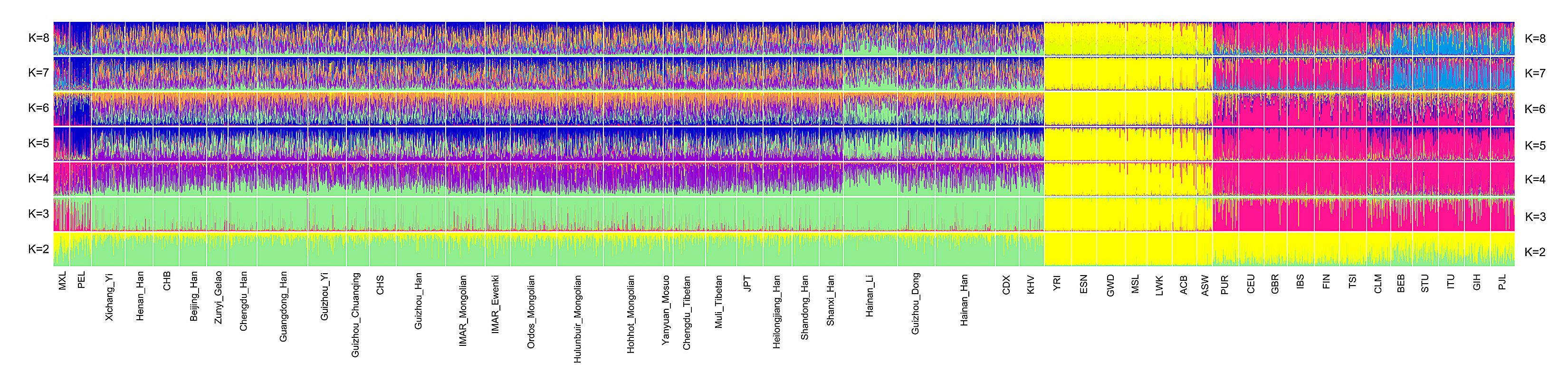


**Figure S2.** Results of STRUCTURE analysis based on raw genotypes of 7 541 individuals with K values set from 2 to 8.
